# Brassinin alleviates cancer cachexia by suppressing diverse inflammatory mechanisms in mice

**DOI:** 10.1002/mco2.558

**Published:** 2024-05-28

**Authors:** Min Hee Yang, Young Yun Jung, Jae‐Young Um, Gautam Sethi, Kwang Seok Ahn

**Affiliations:** ^1^ Department of Science in Korean Medicine Kyung Hee University Seoul South Korea; ^2^ Department of Pharmacology and NUS Centre for Cancer Research (N2CR) Yong Loo Lin School of Medicine National University of Singapore Singapore Singapore

**Keywords:** Adipocyte atrophy, Brassinin, Cancer cachexia, Muscle atrophy, STAT3

## Abstract

Cancer cachexia is a multifactorial condition that contributes to the death of about 20% of cancer patients. It has the potential to cause weight loss, reduction in muscle mass, and loss of fat tissue, significantly lowering the quality of life. Currently, there are no approved drugs for cancer cachexia. Here, we have explored the possible impact of brassinin (BSN) on cancer cachexia under in vitro and in vivo settings. After differentiation, C2C12 and 3T3‐L1 cells were incubated with colorectal carcinoma cells conditioned media or BSN. For preclinical studies, mice were injected with HT‐29 cells followed by intraperitoneal administration of BSN, and muscle and adipose tissues were evaluated by Western blotting and hematoxylin and eosin staining. BSN effectively suppressed muscle atrophy by down‐regulating the levels of Muscle RING‐finger protein‐1 and Atrogin‐1, while also increasing the expression of myosin heavy chain in cachexia‐induced‐C2C12 myotubes. The induction of adipogenesis by BSN prevented adipocyte atrophy in cachexia‐induced 3T3‐L1 adipocytes. We also noted that BSN disrupted the interaction between COX‐2 and signaling transducer and activator of transcription 3 (STAT3) promoter, leading to down‐regulation of STAT3 activation. Moreover, it was found that BSN inhibited weight loss in mice and demonstrated anti‐cachexic effects. Overall, our observations indicate that BSN can attenuate cancer cachexia through diverse mechanisms.

## Graphical abstract

Cancer cachexia is a multifactorial syndrome that is responsible for the death of approximately 20% of cancer patients. It can effectively cause weight loss, skeletal muscle atrophy, and adipose tissue atrophy which can adversely affect the quality of life. However, effective drugs for cancer cachexia are still lacking. In this study, we have investigated whether brassinin (BSN) can affect cancer cachexia under in vitro and in vivo settings. We found that BSN effectively suppressed muscle atrophy by down‐regulating the levels of Muscle RING‐finger protein‐1 and Atrogin‐1, accompanied by increased expression of myosin heavy chain in cachexia‐induced‐C2C12 myotubes. BSN inhibited adipocyte atrophy by inducing adipogenesis in cachexia‐induced 3T3‐L1 adipocytes. We also noted that BSN can down‐regulate signaling transducer and activator of transcription 3 activation. Moreover, it was found that BSN inhibited weight loss in mice and exerted anti‐cachexic actions. Overall, our observations indicate that BSN can attenuate cancer cachexia and might function as a novel agent for the treatment of cancer cachexia.

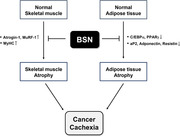

## 1 INTRODUCTION

Cancer cachexia is a condition characterized by uncontrollable weight loss. It significantly impacts patients' quality of life and survival rates by leading to muscle wasting, fat loss, loss of appetite, weight reduction, and anemia.^1^, ^2^, ^3^ The exact causes of this syndrome are not fully known, but it is believed to involve various factors such as the production of inflammatory cytokines, insulin resistance, catabolic factors, negative protein/energy balance, and abnormal metabolism.^4^, ^5^, ^6^, ^7^, ^8^ Inflammatory pathways are closely linked to numerous metabolic disorders, including anorexia, and are key to the underlying mechanisms of cachexia.^9^, ^10^ In recent years, cachexia has been viewed as a systemic inflammatory response that is driven by various cytokines.^11^ Corticosteroids, which are anti‐inflammatory drugs, have been utilized in the treatment of cancer cachexia. In the United States and Europe, these drugs are prescribed to manage anorexia in cachexia patients and have demonstrated effectiveness in preserving weight and improving quality of life. However, their use is limited by the substantial risk of side effects associated with long‐term administration.^12^ Moreover, the advent of anti‐inflammatory medications, such as COX‐2 inhibitors, is beginning to show promising approaches for addressing cancer cachexia.^13^, ^14^, ^15^ COX‐2 inhibitor celecoxib, one of the non‐steroidal anti‐inflammatory drugs, has exhibited significant potential in the treatment of cancer cachexia.^14^, ^15^ Additionally, cachexia can arise as a side effect of chemotherapy, further contributing to poor prognosis.^3^, ^16^, ^17^ In advanced stages of cancer, over half of the patients exhibit symptoms of cachexia, with around 20% of cancer‐related deaths being attributed to this condition.^2^, ^7^

The decline in skeletal muscle mass is attributed to an imbalance between anabolic and catabolic processes, along with the activation of various signaling pathways, including the signaling transducer and activator of transcription 3 (STAT3), forkhead box protein O3, and nuclear factor‐kappa B.^18^, ^19^, ^20^, ^21^, ^22^, ^23^ Notably, the IL‐6/STAT3 pathway may play a vital role in the advancement of cachexia.^19^, ^24^, ^25^ These activated signaling pathways can lead to an increase in E3 ubiquitin ligases, which are crucial in regulating muscle wasting.^26^ Muscle RING‐finger protein‐1 (MuRF‐1) and Atrogin‐1 are examples of ubiquitin ligases involved in muscle breakdown.^27^ The activation of MuRF‐1 and Atrogin‐1 can further promote the ubiquitination of substrates, enhance the degradation by proteasomes, and increase muscle atrophy, including in skeletal muscles, potentially leading to cancer cachexia.^28^, ^29^

Cancer cachexia is associated not only with the loss of skeletal muscle but also with the reduction of adipose tissue. Specifically, it results in a marked decrease in white adipose tissue (eWAT) in addition to skeletal muscle wasting. Adipose tissue loss has been correlated with decreased quality of life and survival time.^30^ Activated atrophy in adipocytes can substantially reduce cell volume and also mitigate the process of new adipogenesis.^2^ Impaired adipogenesis, browning of WAT, and increased lipolysis constitute the key underlying mechanisms of adipose loss in cancer cachexia.^31^, ^32^ Interestingly, C/EBPα and PPARγ have been reported as differentiation factors capable of regulating the process of adipogenesis.^31^, ^33^ In addition, adiponectin, aP2, and resistin can target adipocyte‐specific gene expression.^34^ Therefore, uncovering the mechanisms behind adipose tissue loss is vital for finding innovative therapeutic solutions for cancer cachexia patients.

The potential application of natural agents against cachexia remains an important strategy as they have been found to target various characteristics associated with tumorigenesis.^35^, ^36^, ^37^ Brassinin (BSN), a phytoalexin derived from different cruciferous vegetables has been documented to exert diverse pharmacological actions by acting as an anti‐proliferative, anti‐oncogenic, and anti‐fungal agent.^38^, ^39^, ^40^, ^41^, ^42^ Our team has documented that BSN is capable of influencing the epithelial‐mesenchymal transition process by impacting the PI3K/Akt/mTOR signaling pathways in lung carcinoma.^42^ In addition, BSN can exert an anti‐pleiotropic effect through modulating PI3K/Akt/mTOR and JAK/STAT3 pathways in colorectal cancer.^39^ Furthermore, Kim et al. found that BSN and capsaicin, when used together, could significantly boost the anti‐metastatic effects in prostate cancer.^43^ However, there are no reports existing in the literature regarding the possible impact of BSN in cancer cachexia.

Currently, effective medications for cancer cachexia are scarce, with no treatments specifically approved by the Food and Drug Administration for this condition.^44^ Hence, in this study, the anti‐cachexia effects of BSN were deciphered and it was observed that BSN could attenuate cancer cachexia‐induced muscle and adipose tissue atrophy through diverse molecular mechanisms including inhibition of COX‐2 and STAT3.

## 2 RESULTS

### 2.1 BSN inhibited colorectal carcinoma cells conditioned media‐induced cancer cachexia and COX‐2 expression

**FIGURE 1:**
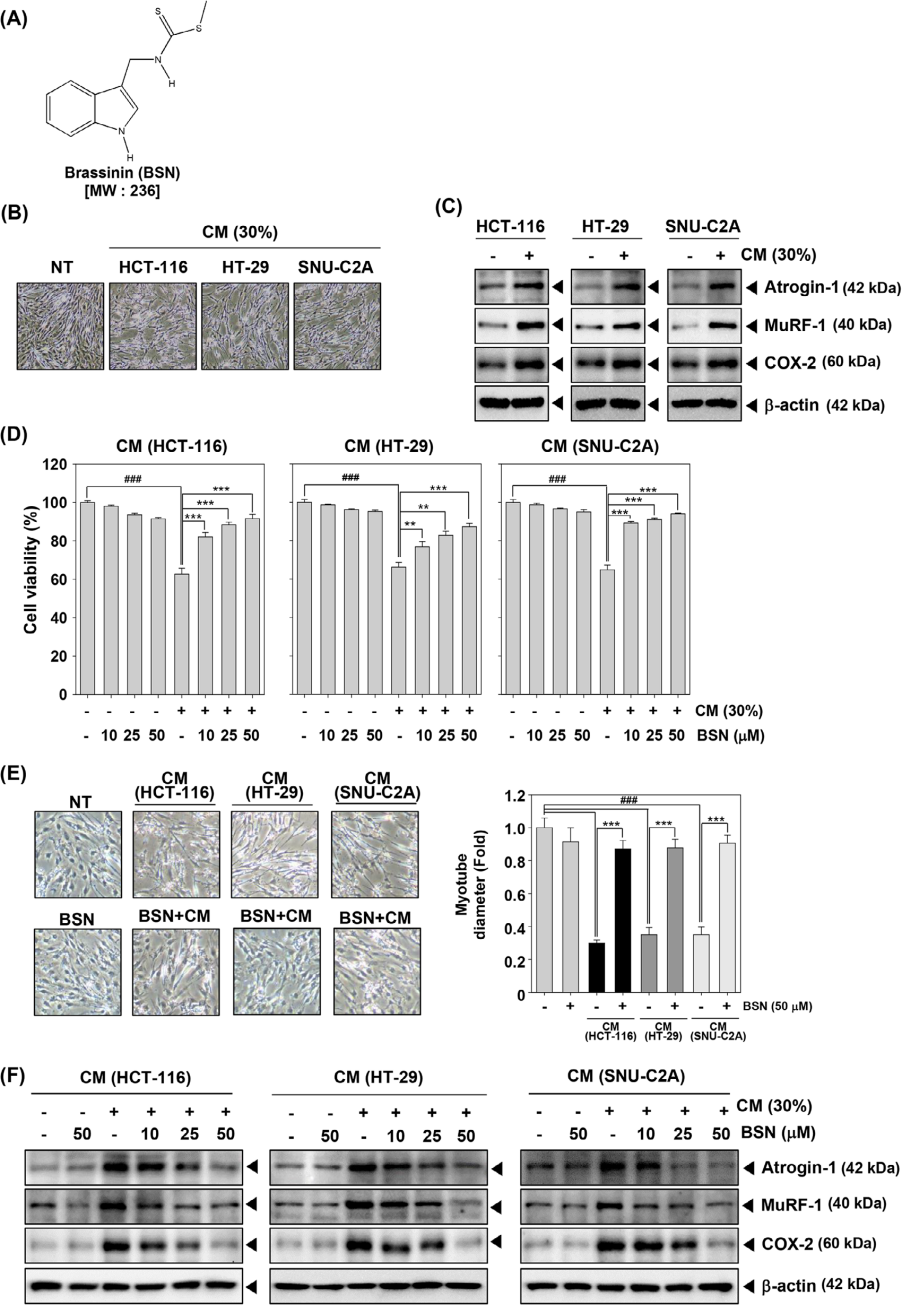
Brassinin (BSN) down‐regulates cancer cachexia‐induced atrophy of muscle cells. (A) The chemical structure of brassinin (BSN). (B) C2C12 cells were incubated with HCT‐116, HT‐29, and SNU‐C2A cells conditioned media (CM) for 24 h. Morphological changes of the cells were detected using a microscope. (C) The cells were treated as described in panel B, and immunoblotting was carried out. (D) Cell viability was determined by MTT assay after incubation with various cancer cells CM (30%) and BSN (0, 10, 25, and 50 μM) for 24 h. Data analysis was performed using SigmaPlot software. (E) Cells were treated with CM (30%) and BSN (50 μM) and morphological changes and myotube diameter were observed. Data represents mean ± SD. ^###^ *p* < 0.001 versus non‐treated cells and ^***^ *p* < 0.001 versus cancer cells CM treated cells. (F) The cells were incubated in panel C and western blotting was done.

Muscle wasting is a principal consequence of cancer cachexia. To assess its effect on muscle atrophy and explore the anti‐cachexia properties of BSN, we first analyzed its impact on myotube atrophy using C2C12 mouse myoblast cells. The chemical structure of BSN is illustrated in Figure 1A. After the differentiation of C2C12 cells, morphological changes were observed. As shown in Figure 1B, differentiation into myotubes generated thick and elongated structures in non‐treated (NT) C2C12 cells. However, myotubes were relatively thin and atrophied upon exposure to conditioned media (CM). The results indicated that HCT‐116, HT‐29, and SNU‐C2A cells CM induced Atrogin‐1, MuRF‐1, and COX‐2 expressions which are known to play roles in regulating muscle atrophy in cachexia (Figure 1C). As depicted in Figure 1D, various colorectal carcinoma CM treatments significantly decreased cell viability, but BSN treatment can attenuate cachexia‐mediated cell death. In addition, BSN reversed CM‐induced myotube atrophy as determined by morphological changes and myotube fiber width (Figure 1E). Moreover, in various colorectal carcinoma cells, CM‐stimulated expression of Atrogin‐1, MuRF‐1, and COX‐2 was suppressed by BSN (Figure 1E). These observations indicated that BSN can significantly reduce cell death by attenuating cancer cachexia.

### 2.2 BSN‐induced expression of myosin heavy chain

**FIGURE 2:**
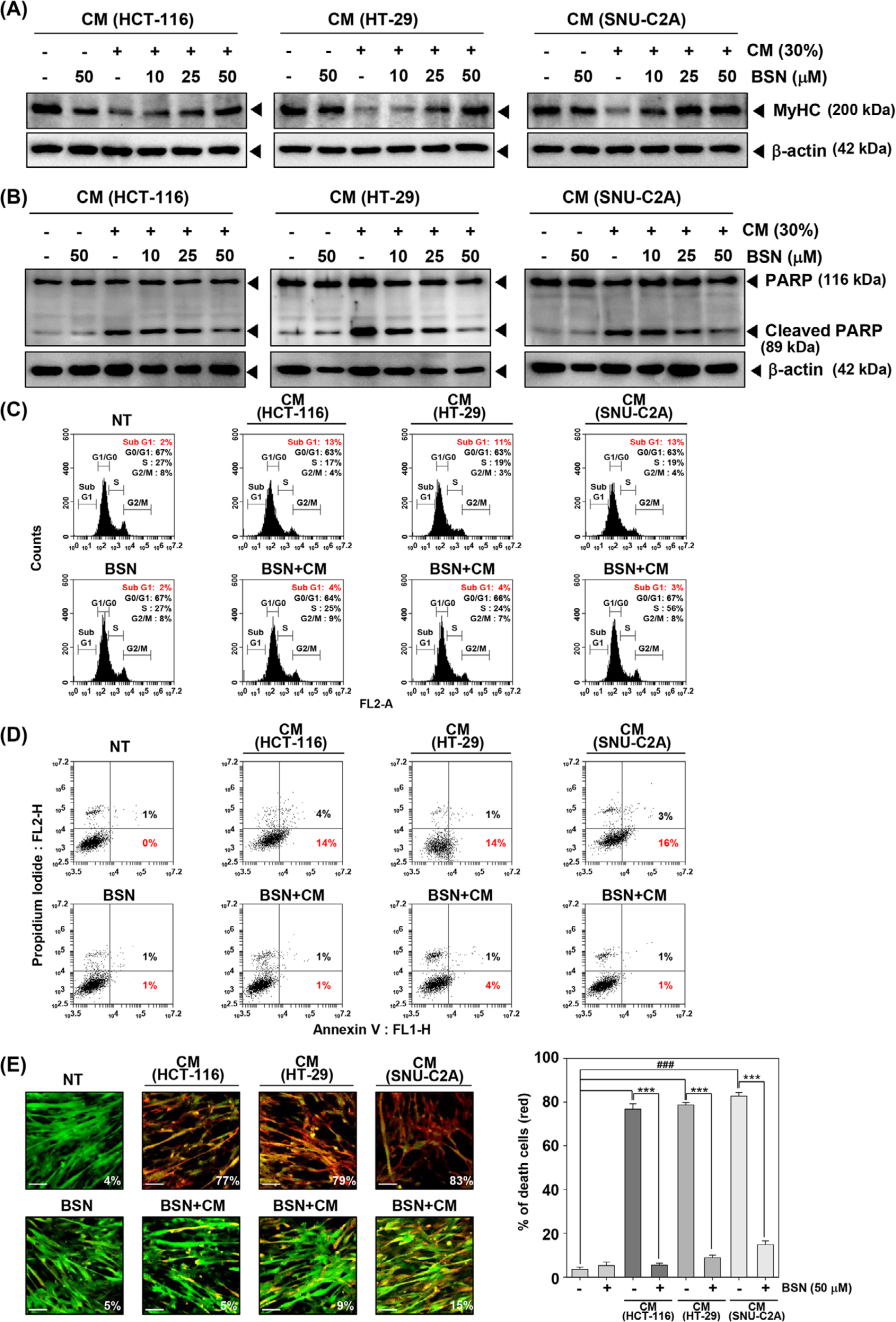
Brassinin (BSN) induces the myosin heavy chain (MyHC) expression and suppresses conditioned media (CM)‐induced apoptosis. (A, B) Representative Western blots showing MyHC and PARP protein expression in C2C12 cells treated with CM (30%) and BSN (0, 10, 25, and 50 μM). (C) The cells were treated with cancer cells CM (30%) and BSN (50 μM) and cell cycle analysis was conducted. The cells were analyzed by BD AccuriTM C6 Plus Flow Cytometer (BD Biosciences, Bec‐ton‐Dickinson) with BD Accuri C6 Plus software. (D) Annexin V assay was done after cells were treated with CM (30%) and 50 μM of BSN. The cells were analyzed by BD AccuriTM C6 Plus Flow Cytometer (BD Biosciences, Bec‐ton‐Dickinson) with BD Accuri C6 Plus software. (E) Live and dead assay was carried out (scale bar: 50 μm). Data represents mean ± SD. ^###^ *p* < 0.001 versus non‐treated cells and ^***^ *p* < 0.001 versus cancer cells CM treated cells.

Given that myosin heavy chain (MyHC) is a key gene product in skeletal muscle, we analyzed the impact of BSN in restoring MyHC expression.^45^ When HCT‐116, HT‐29, and SNU‐C2A cells CM were added to C2C12 myotubes, the level of MyHC was inhibited in comparison to NT cells (Figure 2A). However, BSN was found to restore the levels of MyHC to the normal state. These results indicated that BSN has the potential to modify the expression of skeletal muscle gene products in C2C12 myotubes.

### 2.3 BSN suppressed cancer cachexia‐induced apoptosis

Additionally, we also investigated the ability of BSN to inhibit apoptosis induced by cancer cachexia. Apoptosis is a crucial process that facilitates protein degradation during muscle wasting. It can aid in controlling muscle mass loss that can occur during cachexia.^46^ As shown in Figure 2B, HCT‐116, HT‐29, and SNU‐C2A cells CM induced expression of PARP cleavage and BSN suppressed its expression. CM obtained from various colorectal carcinoma cells induced subG1 phase arrest and early apoptosis, however, BSN alleviated these findings (Figure 2C,D). In addition, CM‐induced cell death was suppressed by BSN as analyzed by using live and dead assay (Figure 2E).

### 2.4 BSN caused adipogenesis and differentiation‐related factors

**FIGURE 3:**
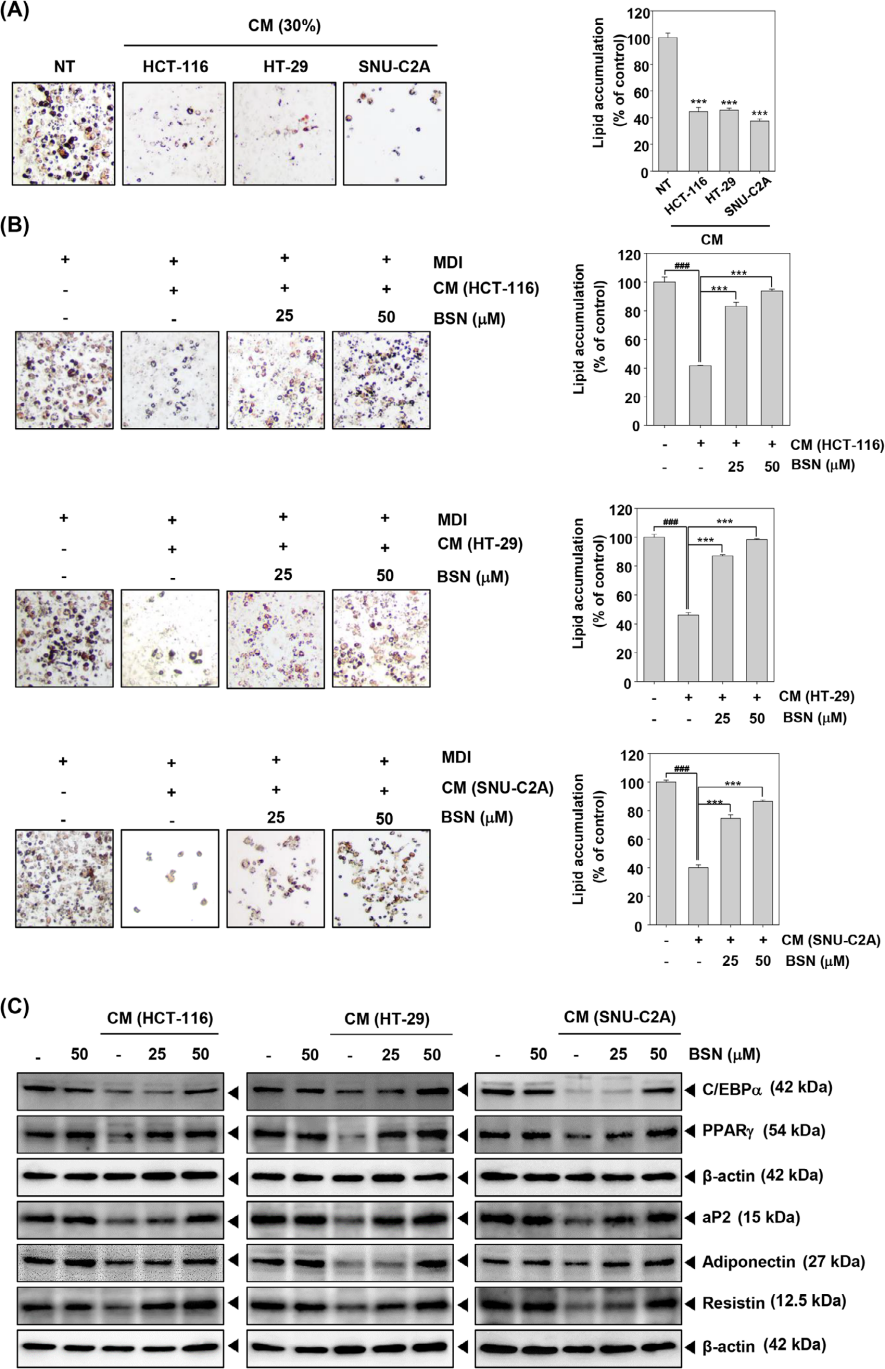
Brassinin (BSN) inhibits cancer cachexia‐induced adipocytes atrophy and induced adipogenesis factors. (A) After differentiation of 3T3‐L1 into adipocytes, the lipid droplets were determined by oil red o staining. (B) The cells were treated with cancer cells conditioned media (CM) (30%) and BSN (0, 25, and 50 μM) for 2 days. ^###^ *p* < 0.001 versus non‐treated cells and ***p < 0.001 versus cancer cells CM treated cells. Data analysis was performed using SigmaPlot software. (C) Western blotting was conducted in 3T3‐L1 cells.

After differentiation, 3T3‐L1 cells were incubated with the CM or media including BSN and insulin for two days. Thereafter, cells were incubated with media containing insulin for four days. We then determined whether HCT‐116, HT‐29, and SNU‐C2A cells CM could induce adipocyte atrophy by oil red o staining. Interestingly, the results of oil Red o staining indicated that CM‐treated adipocytes have fewer lipid droplets than NT cells (Figure 3A). However, BSN enhanced the amount of lipid droplets (Figure 3B). The lipid aggregation was measured by VARIOSKANLUX. Next, we confirmed the potential effect of BSN on adipogenesis. As demonstrated in Figure 3C, CM suppressed C/EBPα, PPARγ, aP2, adiponectin, and resistin expression, whereas BSN increased their expression levels. These results suggested that BSN could target cachexia by causing adipocyte atrophy suppression and inducing adipogenesis in 3T3‐L1 cells.

### 2.5 BSN inhibited STAT3 signaling in C2C12 and 3T3‐L1 cells

**FIGURE 4:**
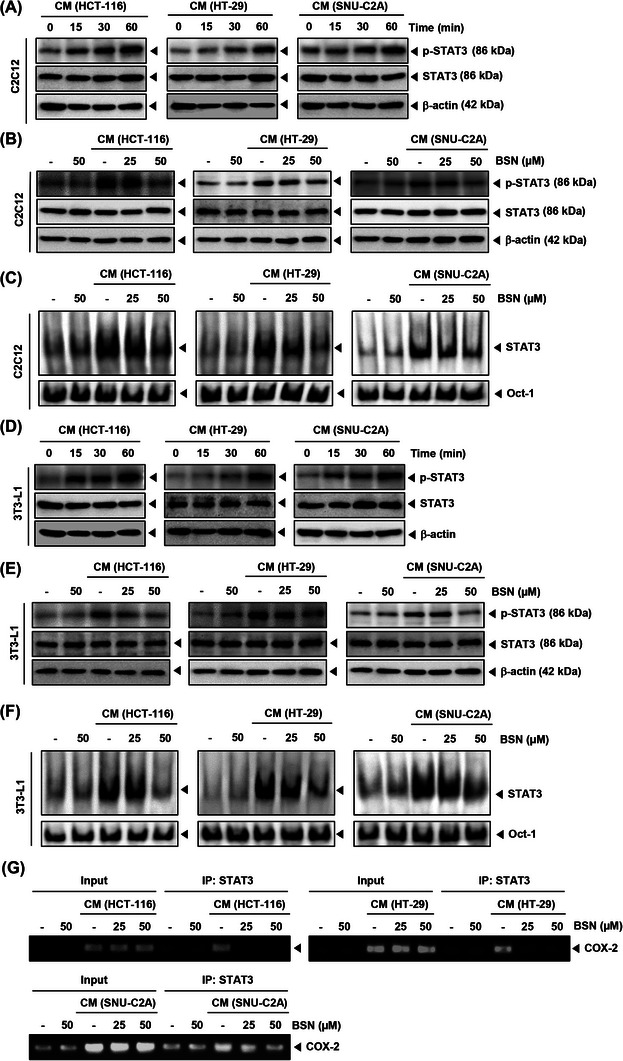
Brassinin (BSN) decreases cancer cachexia‐induced activation of STAT3 in C2C12 and 3T3‐L1 cells. (A) Representative immunoblotting for p‐STAT3 and STAT3 protein in C2C12 cells. (B) C2C12 cells were incubated with BSN (0, 25, and 50 μM) and stimulated with cancer cells CM (30%) for 60 min. p‐STAT3 and STAT3 expression levels were determined by immunoblotting. (C) Electrophoretic mobility shift assay (EMSA) for STAT3 was done after C2C12 cells were treated with BSN and stimulated with conditioned media (CM). (D) 3T3‐L1 cells were treated with various cancer cells CM (30%) for (0, 15, 30, and 60 min). expressions of p‐STAT3 and STAT3 were evaluated by immunoblotting. (E) 3T3‐L1 cells were treated with BSN (0, 25, and 50 μM) and stimulated with cancer cells CM (30%) for 60 min. Western blotting for p‐STAT3 and STAT3 was conducted. (F) 3T3‐L1 cells were treated BSN and stimulated with CM and the levels of STAT3 were examined by EMSA. (G) Chromatin immunoprecipitation (ChIP) assays were performed to analyze the inhibitory effect of BSN on COX‐2 binding to STAT3 promoter.

To evaluate the underlying mechanisms, we first examined STAT3 phosphorylation. Initially, we treated cells with CM for (0, 15, 30, and 60 min) and performed Western blotting for p‐STAT3 in C2C12 cells. As illustrated in Figure 4A, CM induced maximal phosphorylation of STAT3 at the 60 min time point. Interestingly, BSN could effectively down‐regulate the p‐STAT3 expression and its DNA‐binding property, in spite of the development of CM‐induced cachexia (Figure 4B,C). Furthermore, we verified the influence of BSN on 3T3‐L1 adipocytes, and the cells were exposed to CM for various durations (Figure 4D). We noted that CM induced the maximal STAT3 phosphorylation at 60 min and observed that BSN substantially suppressed the levels of STAT3 (Figure 4E,F). These findings demonstrated that BSN could effectively alter STAT3 activation in C2C12 myotubes and 3T3‐L1 adipocytes. Figure 4G demonstrates that BSN interfered with the interaction between COX‐2 and the STAT3 promoter, subsequently inhibiting the expression of various genes that are regulated by STAT3.

### 2.6 BSN suppressed cancer cachexia in xenograft mouse model

**FIGURE 5:**
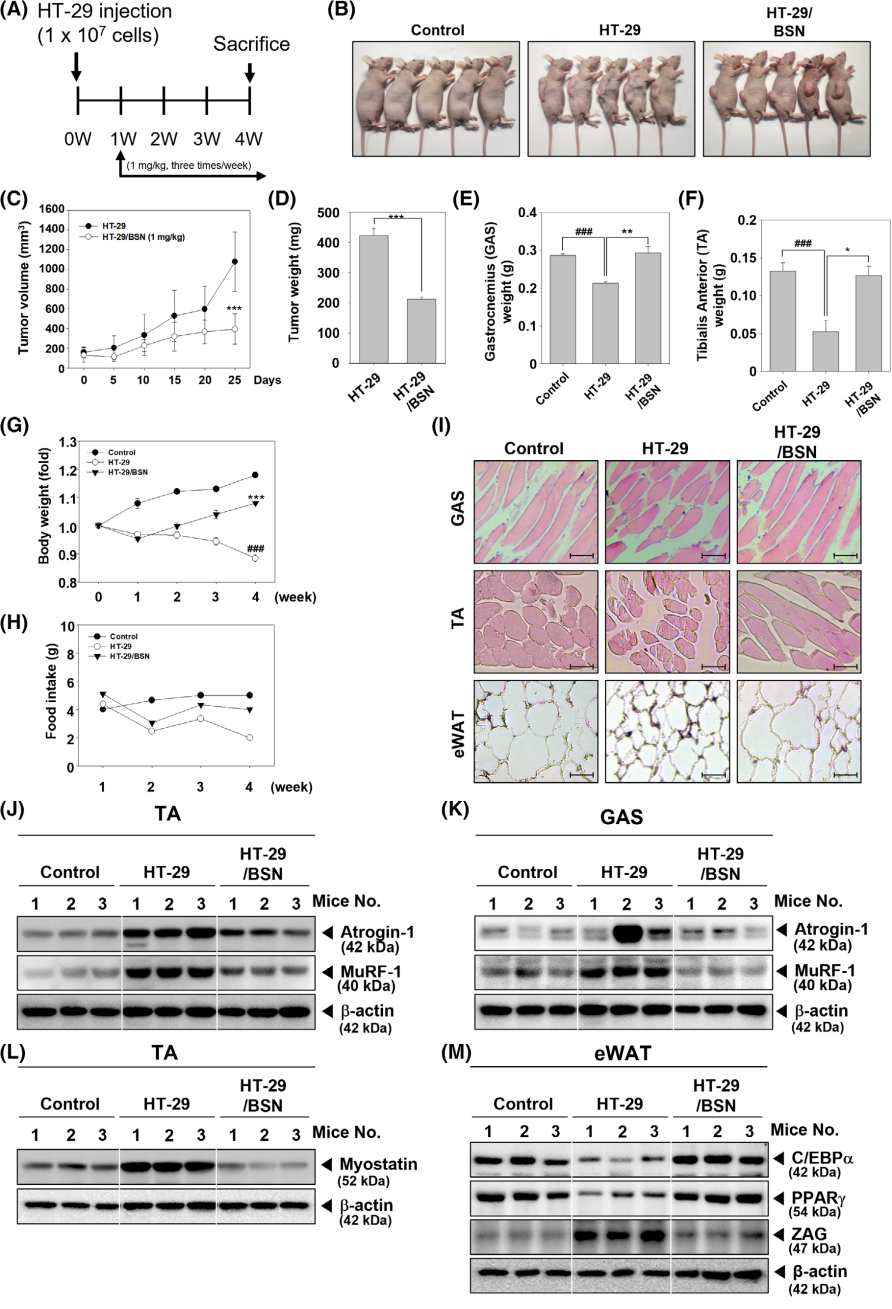
Brassinin (BSN) suppresses cancer cachexia‐induced muscle and adipocyte atrophy in a preclinical model. (A) The schematic representation of in vivo experiment. HT‐29 cells (5 × 10^6^ cells/mouse) were injected subcutaneously and the mice were randomly divided into three different treatment groups (*n* = 6/group). Group I as the control group, group II comprised of mice injected with HT‐29 cells (5 × 10^6^ cells/mice), and group III consisting of mice exposed to BSN (1 mg/kg; intraperitoneal (i.p.) injection: three times a week) after HT‐29 cells injection. The therapy was continued for 4 weeks from the randomization (0 weeks). (B) Representative photographs of mice's appearance. (C) Tumor volume was measured during the experiment. (D–F) Tumor weight, gastrocnemius (GAS), and tibialis anterior (TA) weight were measured on the last day of the experiment. (G, H) Changes in body weight and food intake at the indicated days. Data analysis was performed using SigmaPlot software. (I) GAS, TA, and white adipose tissue (eWAT) were stained to compare cancer cachexia‐induced atrophy (scale bar: 50 μm). (J–M) The expression levels of diverse markers were analyzed.

Next, we further assessed the anti‐cachexia properties of BSN using an HT‐29 xenograft model, which was established to induce cancer cachexia in mice. The experimental schedule is shown in Figure 5A, and graphs indicated that tumor volume rapidly increased in the HT‐29 xenograft group, but was significantly decreased in the BSN‐treated group (Figure 5B,C). There were no dead mice observed during the preclinical experiments. After sacrificing the animals, tumor, tibialis anterior (TA), and gastrocnemius (GAS) were harvested and their weights were measured (Figure 5D–F). The weights of both GAS and TA were decreased in the HT‐29 xenograft group, however, the weight losses were inhibited in the BSN‐treated group. Interestingly, cancer cachexia‐induced mice displayed body weight loss, but BSN treatment was able to significantly recover it (Figure 5G). As shown in Figure 5H, food intake decreased significantly in the HT‐29 xenograft group, but a slight increase was observed in the BSN treatment group. Histological examination of GAS, TA, and eWAT was conducted to examine cancer cachexia‐induced atrophy (Figure 5I). Surprisingly, cachexia was observed and it was accompanied by atrophy of GAS, TA, and eWAT, but it was attenuated by BSN treatment. In addition, as shown in Figure 5J,K, expressions of Atrogin‐1 and MuRF‐1 were induced, however, BSN suppressed the levels of these markers in TA and GAS. Myostatin, which can down‐regulate myogenesis was induced but BSN suppressed its expression (Figure 5L). C/EBPα and PPARγ expression levels were suppressed, but BSN was able to restore their levels (Figure 5M). These findings suggested that BSN could ameliorate cancer cachexia in xenograft mouse models.

## 3 DISCUSSION

**FIGURE 6:**
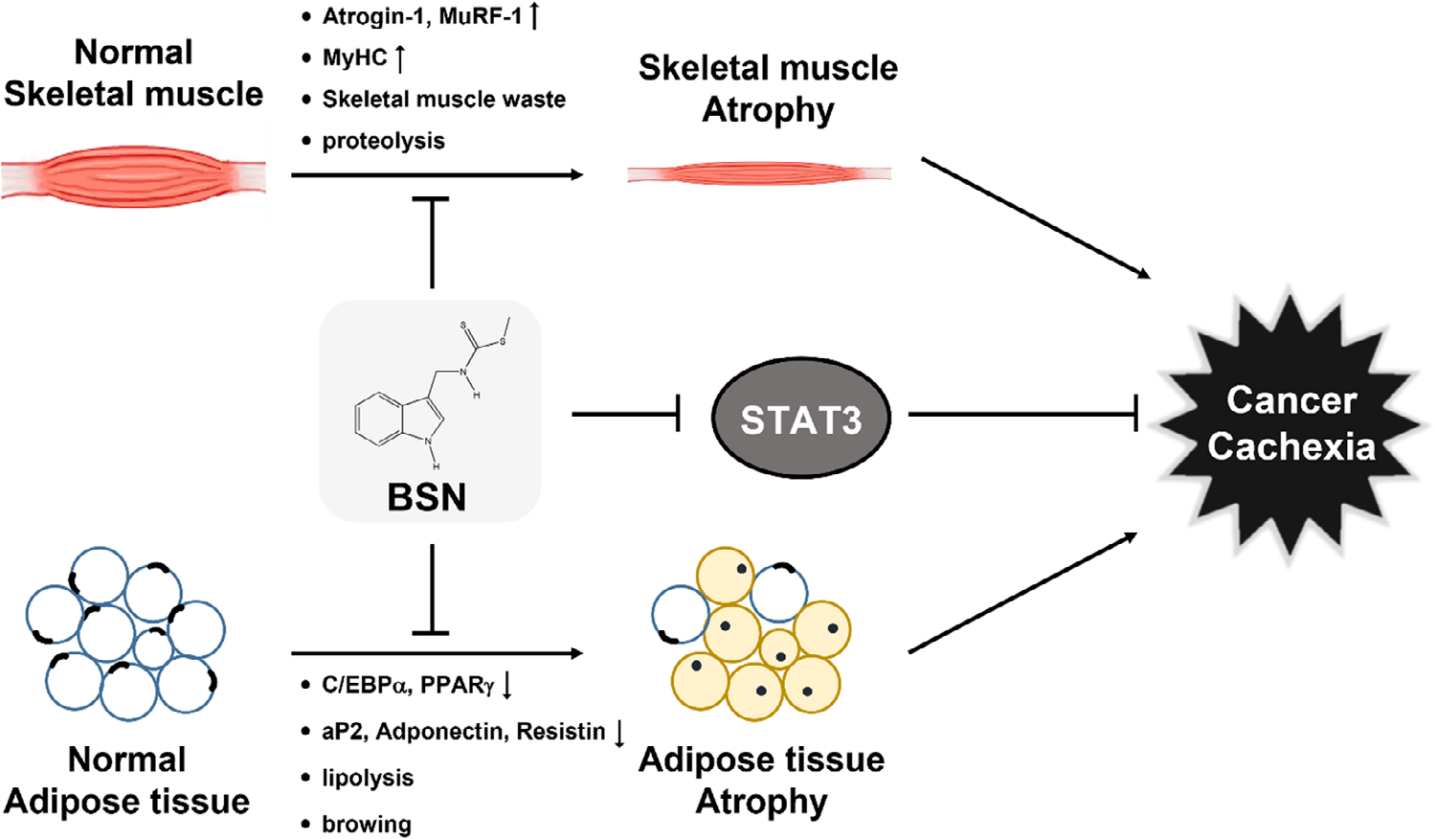
A diagram illustrating the anti‐cachexic properties of brassinin (BSN).

It has been reported earlier that BSN can affect the effectiveness of chemotherapy against several types of cancers.^38^, ^39^, ^40^, ^41^, ^42^, ^43^ Despite the potential of BSN, its effect on cancer cachexia has not been documented previously. Our goal was to investigate the impact of BSN on cancer cachexia and to clarify the mechanisms involved. Weight loss, anemia, asthenia, and anorexia have been established as common symptoms associated with cachexia,^47^, ^48^, ^49^ hence we examined the influence of BSN signaling pathways associated with muscle and adipocyte atrophy. Furthermore, past research has suggested a possible association between COX‐2 and cachexia, with the inhibition of COX‐2 shown to relieve symptoms of cachexia.,^13^, ^14^, ^15^ Building on this, we sought to investigate the effect of BSN on reducing cancer cachexia through the inhibition of COX‐2. The present study provides the first evidence that BSN has the potential to mitigate cancer cachexia and influence the resulting loss of muscle (Figure 6).

Muscle atrophy is recognized as a key event in the development of cancer cachexia.^19^, ^24^, ^25^ The affected patients who are affected by muscle wasting primarily exhibit activation of Atrogin‐1 which belongs to the F‐BOX family.^29^ MuRF‐1 is a ubiquitin ligase that acts in conjunction with Atrogin‐1.^50^ Chen and colleagues have reported that imperatorin can reduce cancer cachexia and muscle loss by adjusting the levels of MuRF‐1, Atrogin‐1, and MyHC.^3^ Based on this, we initially assessed its impact on muscle atrophy induced by cachexia in C2C12 myotubes. We induced cachexia in C2C12 cells using HCT‐116, HT‐29, and SNU‐C2A colorectal carcinoma cells CM. Exposure to CM obtained from different cancer cells induced morphological changes and expression levels of MuRF‐1 and Atrogin‐1 which are representative muscle atrophy markers, but BSN suppressed expression of these proteins in CM‐treated C2C12 cells. We also noted that cancer cachexia down‐regulated MyHC expression, which is a musculoskeletal factor, and BSN also increased MyHC expression. Moreover, the apoptotic pathway is crucial in promoting protein breakdown during muscle wasting.^46^, ^51^, ^52^ Several studies have documented that apoptosis takes place in individuals experiencing cancer cachexia, which results in protein degradation and contributes to muscle atrophy. Therefore, the development of a new drug that can target apoptosis could be a potential strategy to minimize the side effects caused by tumor progression.^46^, ^51^, ^53^ We found that cancer cachexia promoted both apoptosis and tumorigenesis, however, BSN markedly inhibited both these processes. These findings indicated that BSN could suppress cancer cachexia by attenuating muscle atrophy and tumorigenesis through modulating apoptosis.

Adipose dysfunction is also a typical symptom of cancerous cachexia.^2^, ^34^ The loss of adipose tissue in cancer cachexia is mainly attributed to heightened lipolysis, marked by an elevated turnover of both free fatty acids and glycerol, especially when compared to cancer patients who do not experience weight loss.^54^ Adipocyte atrophy can reduce the production of new fat and cell volume.^1^, ^2^ In addition, as evidenced by oil red o staining, CM treatment significantly decreased the number of lipid droplets, however it was increased upon exposure to BSN. Furthermore, cancer cachexia causes a reduction of C/EBPα and PPARγ protein expression, which can function as adipogenesis regulation factors.^31^, ^33^ Adiponectin, aP2, and resistin can regulate the expression of adipocyte‐specific genes.^34^, ^55^ Cancer cachexia also reduced the expression levels of adiponectin, aP2, and resistin, but BSN restored its expression levels. These results suggested that BSN can inhibit cancer cachexia and adipocyte atrophy by hindering the differentiation of adipocytes and increasing adipogenesis factors.

Cancer cachexia involves a variety of pathways, with STAT3 activation being a key factor in skeletal muscle wasting as observed in animal models of cancer cachexia.^56^ Furthermore, the activation of STAT3 can increase the expression of MuRF‐1 and Atrogin‐1, contributing to muscle atrophy.^20^ STAT3 also plays a role in exacerbating cancer cachexia through the promotion of metastasis, immune suppression, and tumor growth.^57^ Therefore, STAT3 may function as a potential molecular target for combating cancer cachexia, with numerous studies exploring the regulation of this condition by inhibiting STAT3.^3^, ^5^, ^7^, ^19^, ^58^ Chen et al found that cryptotanshinone prevented muscle wasting by inhibition of STAT3 activation in cancer cachexia.^58^ Alantolactone suppressed muscle atrophy and adipocyte atrophy by inhibiting the STAT3 signaling pathway in cancer cachexia‐induced C2C12 and 3T3‐L1 cells and experimental mouse model.^5^ We demonstrated that BSN could effectively down‐regulated STAT3 activation in C2C12 myotubes treated with CM. BSN also inhibited activation of STAT3 in 3T3‐L1 adipocytes treated with CM. Thus, the inhibitory effect of BSN on cancer cachexia could be mediated through modulation of the STAT3 pathway.

Inflammatory pathways are crucial in the fundamental mechanisms that drive cachexia.^9^, ^10^ Recently, studies to develop cancer cachexia treatment by targeting different inflammatory pathways have gained prominence. COX‐2 is the inducible isoform of the cyclooxygenase enzyme family, which can be targeted by anti‐inflammatory drugs.^59^, ^60^ Past research has highlighted celecoxib, a COX‐2 inhibitor, as having a promising role in treating cancer cachexia.^13^, ^14^, ^15^ For example, in the mouse colon tumor model, both IL‐6 and parathyroid hormone‐related proteins, which are upregulated by COX‐2, were implicated in the onset of cachexia. Furthermore, COX‐2 inhibitors have been shown to suppress the production of parathyroid hormone‐related proteins.^61^ We also observed in this study that colorectal carcinoma CM up‐regulated COX‐2 expression and BSN suppressed the CM‐induced COX‐2 expression. Furthermore, STAT3 has been identified as a crucial factor in inflammation‐related cancers.^62^, ^63^, ^64^ Previous studies have shown that inhibiting STAT3 activation can decrease COX‐2 expression.^65^ Intriguingly, our findings also revealed that BSN could inhibit the activation of STAT3 and prevent STAT3 from binding to the COX‐2 promoter. Based on these outcomes, we conclude that the anti‐cachexia effect of BSN is linked to a reduced inflammatory response through the modulation of STAT3 activation.

We also confirmed the inhibitory effect of BSN on cancer cachexia under in vivo settings. By establishing a xenograft model through the subcutaneous injection of HT‐29 cells into mice, we were able to study cancer cachexia and observed significant weight loss in the muscle tissues. In addition, increased levels of MuRF‐1, Atrogin‐1, and Myostatin were also observed. However, upon BSN treatment, the weight loss and muscle atrophy were markedly reduced. Adipose tissue eWAT attenuated the expression of adipogenesis and adipogenic differentiation factors in mice. However, adipocyte atrophy was substantially restored in mice treated with BSN.

The current study has shown, for the first time, that BSN is capable of mitigating cancer cachexia, effectively reversing muscle and adipocyte atrophy in models induced with cancer cachexia. Therefore, our findings suggest that BSN holds promise as a new drug candidate for the treatment of cancer cachexia. Moreover, BSN could act as a promising therapeutic option for managing cancer‐related complications, potentially enhancing overall survival rates. While our study provides valuable insights into the potential impact of BSN on cancer cachexia, it's essential to acknowledge certain limitations. First, although we utilized in vitro models (C2C12 and 3T3‐L1 cells), they may not fully recapitulate the complex microenvironment and interactions present in vivo. Thus, findings from these models should be interpreted cautiously. Second, preclinical studies involving mice injected with HT‐29 cells followed by BSN administration were conducted with a limited sample size. This may affect the robustness and generalizability of the observed effects. Third, while we provided mechanistic insights into the effects of BSN on muscle and adipose tissue atrophy, our understanding of the precise molecular pathways involved may be incomplete. Further elucidation of these mechanisms is warranted. Finally, the long‐term effects and safety profile of BSN, particularly with prolonged administration, remain unclear. Hence, comprehensive studies assessing potential adverse effects and toxicity are warranted.

## 4 MATERIALS AND METHODS

### 4.1 Reagents and cell lines

BSN (purity: ≥98%) was purchased from LKT Laboratories and dissolved in DMSO. Mouse myoblast C2C12 and mouse embryo fibroblast 3T3‐L1 cells were purchased from the American Type Culture Collection. C2C12 cells were cultured in a Dulbecco's Modified Eagle Medium (DMEM)/high glucose medium containing 10% fetal bovine serum (FBS) and penicillin‐streptomycin antibiotics (1%). To facilitate the differentiation of C2C12, the cells were incubated with DMEM high glucose medium containing 2% horse serum for 3 days. 3T3‐L1 cells were cultured in a DMEM medium containing 10% FBS and 1% penicillin‐streptomycin‐glutamine. To facilitate their differentiation, cells were incubated with MDI media IBMX (500 μM), Dexamethasone (1 μM), and Insulin (1 μg/mL) for 3 days. Cell conditions were maintained at 37°C in 5% CO_2_ conditions.

CM from colorectal carcinoma cells was collected 1 day after changing the medium.^7^

### 4.2 MTT assay

C2C12 cells (1 × 10^4^ cells/well) were seeded and incubated until confluency reached 90%. The medium was thereafter changed to facilitate differentiation for 3 days. The cells were treated with CM (30%) obtained from colorectal carcinoma cells or BSN (0, 10, 25, and 50 μM) and an MTT assay was performed to analyze the viability as described earlier.^66^

### 4.3 Western blot analysis

C2C12 and 3T3‐L1 cells (5 × 10^5^ cells/well) were treated according to the specified conditions. Subsequently, Western blot analysis for various markers was performed, following previously described methods.^67^, ^68^ Antibodies of Atrogin‐1: sc‐33782, MuRF‐1: sc‐398608, Cleaved PARP: sc‐56196, C/EBPα: sc‐61, PPARγ: sc‐7196, aP2: 2120s, Adiponectin: 2789s, Resistin: sc‐16117, p‐STAT3: 9145s, and Myostatin: sc‐134345 were purchased from Santa Cruz Biotechnology and Cell Signaling Technology (1:3000).

### 4.4 Cell cycle analysis

C2C12 cells (5 × 10^5^ cells/well) were treated with CM (30%) and BSN (0, 10, 25, and 50 μM). The cell cycle was then analyzed using previously reported methods.^69^, ^70^ The analysis was carried out using a BD AccuriTM C6 Plus Flow Cytometer equipped with BD Accuri C6 Plus software.

### 4.5 Annexin V assay

C2C12 cells (5 × 10^5^ cells/well) were treated with CM (30%) and BSN (0, 10, 25, and 50 μM). To validate apoptosis, Annexin V assay was conducted following the previously outlined procedure.^39^ The cells were analyzed by BD AccuriTM C6 Plus Flow Cytometer with BD Accuri C6 Plus software.

### 4.6 Live and dead assay

C2C12 cells were treated with CM (30%) and BSN (0, 10, 25, 50 μM). Subsequently, a live and dead assay was conducted to visualize dead cells. The fluorescence signals were captured using an Olympus FluoView FV1000 confocal microscope.

### 4.7 Oil red O assay

3T3‐L1 cells were cultured in MDI media and then the adipocyte cells were treated with 1 μg/mL of insulin‐containing CM (30%) derived from cells with BSN (0, 25, and 50 μM) for 2 days. The assay was performed as described earlier.^71^

### 4.8 Electrophoretic mobility shift assay

To verify whether BSN can inhibit the STAT3‐DNA binding capability, an electrophoretic mobility shift assay was carried out for STAT3, with Oct‐1 serving as the loading control, following previously established protocols.^72^, ^73^

### 4.9 Chromatin immunoprecipitation assay

After the initial treatment with CM and BSN, C2C12 cells were subjected to chromatin immunoprecipitation assay, following methods described previously.^74^

### 4.10 Experimental protocol and ethical statement

Six‐week‐old athymic nu/nu male mice with a body weight ranging from 18 to 23 g were obtained from JA BIO and housed with a standard laboratory diet (Purina) and water ad libitum. After injection of HT‐29 cells (5 × 10^6^ cells/mouse), tumor diameters were measured using a digimatic caliper. When tumors reached 0.25 cm in diameter, the 18 mice were randomly divided into three different treatment groups (*n* = 6/group). Group I served as the control group, group II comprised of the mice injected with HT‐29 cells (5 × 10^6^ cells/mice) and exposed to PBS (intraperitoneal (i.p.) injection: 3 times a week), and group III consisted of mice exposed to BSN (1 mg/kg; i.p. injection: 3 times a week) after HT‐29 cells injection. The therapy was continued for 4 weeks from the randomization (0 weeks). The experimenter was not blinded to whether the animal received BSN or PBS injection. Bedding and water were replaced weekly, and feed was changed weekly to monitor consumption. The mice were euthanized 3 days after the final therapy, and tissues were processed as described previously.^7^

### 4.11 Hematoxylin and eosin staining

The mice tissues were processed and hematoxylin and eosin staining was done as elaborated previously.^7^

### 4.12 Statistical analysis

The results were presented as means ± standard deviation, and statistical analysis of multiple comparisons was conducted using analysis of variance with Bonferroni's test. A *p*‐value of 0.05 or less was considered statistically significant. All the results shown are representative of three independent experiments.

## AUTHOR CONTRIBUTIONS

M.H.Y.: Conceptualization, formal analysis, and writing‐original draft. Y.Y.J.: Conceptualization and methodology. J.‐Y. U.: Conceptualization and visualization. G.S.: Writing‐original draft and supervision. K.S.A.: Conceptualization, formal analysis, and supervision.

All the data was generated in‐house and no paper mill was used. All authors agree to be accountable for all aspects of work ensuring integrity and accuracy. All authors have read and approved the final manuscript.

## CONFLICT OF INTEREST STATEMENT

The authors declare no conflict of interest.

## ETHICS STATEMENT

All procedures involving animals were reviewed and approved by the Kyung Hee University Institutional Animal Care and Use Committee [KHSASP‐20‐513] (approval date: November 20, 2020).

## Data Availability

The datasets used and/or analyzed during the current study are available from the corresponding author upon reasonable request.
